# Editorial: Decoding complexity: genomic, epigenomic, and environmental dynamics in developmental and neurogenetic disorders

**DOI:** 10.3389/fgene.2025.1724013

**Published:** 2025-10-22

**Authors:** Alessandro Fiorenzano, Luísa Azevedo

**Affiliations:** ^1^ Department of Molecular Medicine and Medical Biotechnology, University of Naples “Federico II”, Naples, Italy; ^2^ Department of Experimental Medical Science, Developmental and Regenerative Neurobiology, Wallenberg Neuroscience Center, Lund Stem Cell Center, Lund University, Lund, Sweden; ^3^ Unit for Multidisciplinary Research in Biomedicine (UMIB), School of Medicine and Biomedical Sciences (ICBAS), University of Porto, Porto, Portugal; ^4^ Laboratory for Integrative and Translational Research in Population Health (ITR), University of Porto, Porto, Portugal

**Keywords:** neurodevelopment, genetics, epigenetics, DNA methylation, variant

## Introduction

With the advance of sequencing methodologies in the last decades, a high number of variants associated to genetic diseases have been identified (Bick et al., 2024). This is accompanied with the identification of novel genotype-phenotype associations and a deeper understanding of the epigenetic mechanism associated to human disease (Farsetti et al., 2023; Zoghbi and Beaudet, 2016). In the Research Topic Decoding Complexity: Genomic, Epigenomic, and Environmental Dynamics in Developmental and Neurogenetic Disorders, we aimed to bring together studies that have explored these distinct dimensions, either isolated or in combination. The five studies of this Research Topic have successfully addressed this objective. Three of the studies focus on the report of disease-associated genetic variants (Boyarchuk et al., Yan et al., Liang et al.) and two (Huang et al., Mascheroni et al.) explore the role of epigenetic modifications.

## Genetic alterations in developmental genes

The number of variants related to developmental processes has continuously increased as a response of a global effort to understand the molecular basis of associated diseases. A clear example is the study of Boyarchuk et al., which reported the results obtained by applying whole-exome sequencing (WES) on 90 children with developmental delay. The authors found pathogenic or likely pathogenic variants in 27.8% of cases, and the percentage of variants of unknown significance reached 7.8%. Overall, the study highlights that the positive and inconclusive cases guided a revision of the diagnosis or management plan in about one-third of the cases, emphasizing the relevance of WES in clinical decisions.

Yan et al. have identified a pathogenic variant (c.415G>A, p. Gly139Arg) in the *KCNK4* gene in a pediatric patient with EFS+, neurodevelopmental abnormalities and hypertrichosis. The study was performed in trio-based whole-exome sequencing and the variant was found to be *de novo*. The pathogenicity of the missense variant was accessed by several layers of information despite the absence of functional studies. According to the authors, the identification of this variant expands the phenotypic spectrum associated to the *KCNK4* gene, suggesting it as a novel candidate gene of EFS+.

Liang et al. documented the results of a study involving two patients carriers of pathogenic variants in the *NR4A2* gene (c.994G>C, p. Val332Leu, and 2q23.3-q24.2 deletion), combined with a review of 19 previously reported NR4A2-related cases offering a broader perspective of the clinical pathogenicity associated with this gene. The authors observed a potential therapeutic benefit through levodopa therapy, which has resulted in substantial clinical improvements of the intellectual development and language proficiency. However, they also acknowledge some limitations of their study, namely, the need for multicenter collaborative studies to enhance statistical power.

## Epigenetic alterations in developmental genes

Epigenetic regulation—including mechanisms such as DNA methylation, histone modification, and non-coding RNA activity—is increasingly recognized as a key interface between genetic predisposition and environmental influences in shaping neurodevelopmental trajectories. These molecular processes do not alter the genetic code itself but modulate gene expression in response to developmental cues, stress, nutrition, or environmental exposures. During early life, when brain plasticity is at its peak, such epigenetic mechanisms are essential for orchestrating neuronal differentiation, synaptic formation, and circuit refinement. Disruptions in these finely tuned regulatory processes can have lasting effects on motor, cognitive, and emotional development. Two recent studies provide novel evidence linking DNA methylation (DNAm) alterations to early brain function and motor development.

In the first study, Huang et al. conducted a genome-wide DNA methylation analysis in children with and without Developmental Coordination Disorder (DCD)—a condition affecting 5%–6% of school-aged children. They identified 416 differentially methylated probes (DMPs) and several differentially methylated regions (DMRs), revealing that methylation levels at FAM45A, FAM184A, SEZ6, and GPD2 were significantly associated with motor performance. These findings suggest that specific epigenetic signatures may influence motor coordination and could serve as biomarkers for early DCD detection and intervention.

Complementing these findings, Mascheroni et al. explored PIEZO1 DNA methylation in infants with various neurodevelopmental disorders (NDs). PIEZO1 encodes a mechanosensitive ion channel central to brain mechanotransduction. Assessing 15 CpG sites across the gene in 24 ND and 22 typically developing infants, the authors found that PIEZO1 hypomethylation was characteristic of the ND group. Such reduced methylation may lead to increased Piezo1 expression, potentially altering the mechanical properties of developing brain tissue and contributing to early dysfunction. The study positions PIEZO1 DNAm as a potential early epigenetic marker of neurodevelopmental risk.

Together, these studies highlight DNA methylation as a shared molecular mechanism linking environmental exposures, gene regulation, and early neurodevelopmental outcomes. They underscore the potential of epigenetic biomarkers—including both gene-specific (PIEZO1) and genome-wide signatures (FAM45A, SEZ6, etc.)—for advancing early diagnosis, risk stratification, and targeted interventions in children at risk for motor and neurodevelopmental disorders.
